# Testing for fertility stalls in demographic and health surveys

**DOI:** 10.1186/1478-7954-9-59

**Published:** 2011-12-08

**Authors:** Michel L Garenne

**Affiliations:** 1 Institut de Recherche pour le Développement, UMI Résiliences, Paris, France; 2 Institut Pasteur, Unité d'Epidémiologie des Maladies Emergentes, Paris, France; 3 Witwatersrand University, School of Public Health, Johannesburg, South Africa

**Keywords:** Demographic transition, Fertility decline, Fertility stall, Statistical methods, Linear regression, Logistic regression, Poisson regression, sub-Saharan Africa

## Abstract

This study compares two methods for testing fertility trends and fertility stalls using Demographic and Health Surveys data. The first method is based on linear regression and uses the equivalence of period and cohort estimates with the same cumulative fertility at age 40, the same number of births, and the same distribution of women by parity. The second method is based on logistic regression. It assumes that the age pattern of fertility is constant over short periods of time. Both methods were applied to fertility trends in several African countries (Ghana, Kenya, Madagascar, Nigeria, Rwanda, Senegal, Tanzania, and Zambia). The two methods were found to predict similar values of cumulative fertility, to produce consistent slopes, to document fertility trends the same way, and to characterize fertility stalls with similar statistical evidence. They can also be used to refute apparent fertility stalls obtained when comparing two point estimates from two successive surveys.

## Introduction

Fertility decline from high values typical of natural fertility to near- or below- replacement fertility is a quasi-universal phenomenon. Fertility decline started in almost all countries in the world at some point in the 19^th ^or 20^th ^century with only a few exceptions, and the fertility transition finished (or was almost finished) in a majority of countries by 2010. The United Nations Population Division anticipates that fertility will continue to decline in the next 40 years in the world as a whole. However, the fertility transition will not be completed by 2050 in countries that are currently classified as "high fertility, " most of them in sub-Saharan Africa. In these countries, one expects a total fertility rate (TFR) of 2.8 children per woman, whereas elsewhere fertility will be below replacement, with TFR equaling 1.8 by 2050 [1]. The transition from high levels (i.e., five to eight children per woman) to low levels of fertility (i.e., two or fewer children per woman) is usually continuous and smooth and spans approximately 60 years, or two generations. Occasionally, this transition can occur much faster (over 15 to 25 years) or slower (over a century or more). A typical example of a smooth fertility transition in Europe is Sweden, where the core of the transition occurred between 1870 and 1930 when the TFR dropped from 4.4 to 1.7 children per woman (corresponding cohorts are 1840 to 1900). Similar changes were found throughout Europe, with the exception of France [2,3].

However, in some cases the transition is not continuous, and long periods of fertility stalls have been documented. A typical case of fertility stall is that of Argentina, where fertility dropped from 7.0 children per woman in 1895 to 3.2 children per woman in 1947 and stayed at the same level for about 30 years before resuming its path in recent years towards replacement fertility [4].

Sub-Saharan Africa is the continent that has started its fertility transition most recently. Fertility levels are still high but tended to decline since the 1970s, and declines occurred even earlier in urban areas of the most advanced countries. Most documented cases of fertility decline in Africa seem to be smooth and continuous, although it has been argued that fertility has stalled in a number of countries [5-16]. A comparison of these various studies shows that authors disagree whether or not fertility has stalled in the same countries [13]. These discrepancies come from differences in the case definition of fertility stalls and different statistical testing methods (or sometimes the lack of statistical testing methods) [17,18].

The aim of this paper is to present and compare two simple methods of analyzing slopes of fertility decline and testing fertility stalls. The first method is based on a demographic reconstruction of synthetic cohorts, and the second is based on a direct analysis of fertility rates. This work is a follow-up of earlier work on the same topic [19-24].

## Data and methods

### Demographic and health surveys data

The Demographic and Health Surveys (DHS) are standard surveys based on representative samples of national populations. Among other information, they provide maternity histories for women aged 15 to 49 with details on the date of each birth and on the age of the mother at time of each birth. This is enough information to compute person-years lived and births by age and time period and therefore age-specific fertility rates by period for the years preceding the survey. Note that since these maternity histories are closed cohorts, these calculations can be done with basic tabulation and simple tools such as a spreadsheet or basic computer programming. The only limitation of this type of information is the truncation effect: one knows the fertility "x" years ago only up to women aged "50 - x" years, because women who were interviewed were required to be under 50 years of age. Therefore, we limited our analysis to fertility up to age 40 and for the 10 years preceding each survey for which information is complete. This truncation of retrospective surveys is inevitable; for example, a woman who gave birth nine years ago at age 41 was not interviewed, as she was 50 at the time of the survey. This effect is better explained in other documents [20,22]. DHS record all births that occurred prior to the survey, starting at age 12 years. Cumulative fertility up to age 40 is noted as TFR(40) in this study and represents the average number of children ever born per woman from age 12 to age 40 years, which represents about 90% of the total fertility up to age 50 (the classic TFR).

### Merging data from several surveys in the same country

If several surveys were available in a country, events and person-years were cumulated in order to provide annual fertility rates for longer periods. The cumulating of several surveys reduces fluctuations due to sample size and on average tends to compensate for minor biases associated with sampling. When displayed in a figure, estimates of cumulative fertility by age 40 grouped by two-year period tend to be quite regular and reveal the major trends in fertility, whether increasing, decreasing, or remaining steady. Of course, only formal statistical testing of slopes allows one to demonstrate an increase in fertility, a fertility decline, or a fertility stall. This method has been explained in more details in other documents [20,22,23].

### Surveys used

For this study, we used the following DHS: Ghana: 1988, 1993, 1999, 2003, 2008; Kenya: 1989, 1993, 1998, 2003, 2008; Madagascar: 1992, 1997, 2003; Nigeria: 1990, 1999, 2003, 2008; Rwanda: 1992, 2000, 2005; Senegal: 1986, 1993, 1997, 2005; Tanzania: 1991, 1996, 1999, 2004, 2007; Zambia: 1992, 1996, 2001, 2007.

All computations were done separately for urban and rural areas, since the trends were often divergent at the beginning of the transition, with an earlier decline in urban areas while fertility continued to rise or stagnated in rural areas.

### Case definition of fertility stall

The criteria used for defining a fertility stall were similar to those proposed by Gendell (1985): fertility decline must have started for some years, then the decline must stop for a few years, and if the stall had come to an end, the fertility decline must have resumed. In terms of slopes, this requires an initial period with a significant negative slope, a second period with a net zero or positive slope, a significant change in slopes between the first and second periods, and, when applicable, a third period with a significant negative slope, with a significant change in slopes between the second and third period (p < 0.05 using 2-tailed tests). We used only linear trends for testing the changes in slopes, since these summarize well the changing slopes and are easy to compute. Note that in any country, changes in total fertility rates can almost always be approximated by linear trends over monotonic periods. The knots defining monotonic periods were chosen visually after plotting the yearly cumulative fertility on a graph and then fine-tuned by computing the intersection points of the two regression lines. The slopes were calculated one by one, on each monotonic segment, with the same linear trend.

### Point estimates versus slopes

Our methods focused on slopes computed over periods for which annual fertility rates were available. These methods are far more stable than simply comparing point estimates. For example, in a DHS based on 6, 000 women, a TFR of 3.50 over the three years preceding the survey could be given with a confidence interval of 3.15 to 3.85 (about 0.25 due to sample size and 0.10 due to design effect). If two surveys are available five years apart, it is almost impossible to test a trend from those two points, unless the difference is very large (> 0.50). Even if the second survey indicates a TFR of 3.10, one cannot rigorously conclude whether fertility declined or stayed constant. The testing of slopes is very different, since it includes all points over the period covered, 10 years before each survey, totaling 15 years if two surveys are available. Furthermore, merging datasets for computing slopes allows one to smooth out erratic values of point estimates: these erratic fluctuations include fluctuations due to sample size and design effect, so that a simple test is enough to prove the slope or the changing of slopes.

### Method 1 for testing changing slopes: demographic approach (linear regression)

The first method used for testing slopes and changes in slopes of fertility trends follows a demographic approach and uses the property of period fertility rates. The concept of TFR is abstract and refers to what is called a "synthetic cohort." In other words, it computes what would be the cumulative fertility of a real cohort if it had the same age-specific fertility rates as those observed over a given period. Here, of course, one ignores mortality, as if all women survive up to age 40, as one would do in a real cohort of women who already reached age 40. One could test the trends in period cumulative fertility (period TFR) as if they were trends in cohort cumulative fertility (i.e., equal to completed family size) with the same level and the same number of births. For example, a period TFR(40) of 5.0 based on 1, 000 births is considered to be equivalent to a cohort cumulative fertility of 5.0 among 200 women, who would have had 1000 births by age 40. Testing trends in cohort fertility therefore requires the distribution of completed family size by parity. As in the real world, when an average completed family size is 5.0, the sample includes women with 0, 1, 2 ... 16+ children ever born, with an average of 5.0. Here the period TFR(40) was simply distributed accordingly, by assuming that at the same level of cumulative fertility, the distribution of women by parity was the same in a period and in a cohort (from 0 to 16+ children ever born). This procedure allows one to obtain a direct measure of the slope and its variance, based on individual women, as one would do in a cohort.

In practice, in Method 1 one proceeds the following way. First, one computes the cumulative fertility, TFR(40), from age-specific fertility rates by single calendar year and five-year age group. Then, one computes the corresponding number of women in the synthetic cohort. These women are distributed by parity using a simple relationship linking the proportion of women with (i) children to the completed family size. These relationships were computed from cohort data using the same DHS, from parity 0 to parity 16+. Then, the sample is analyzed as a cohort sample, and cumulative fertility is related to time in a straightforward linear regression:

$$\text{TFR}\left(40\right)=\text{Constant}+\text{B}\times \text{Time}$$

The model allows us to calculate cumulative fertility by year after linear fitting and the precise fertility trend, to provide confidence intervals for slopes (positive, negative, or zero), and to test for changing slopes using standard Student T-tests. This method requires no hypothesis other than the equivalence between period and cohort, which is the rationale for computing period fertility rates. A regression is also calculated for each monotonic period.

### Method 2 for testing changing slopes: statistical approach (logistic regression)

The second approach focuses on age-specific fertility rates and is based on the fact that women are likely to have only one or no delivery over a period of one year, depending on age and period. Therefore, the method chosen is a linear-logistic model, or logistic regression, where the dependent variable is 1 for a birth and 0 for no birth, and the weights are proportionate to the exact person-years lived over the period. The age pattern of fertility is complex and not easy to parameterize, so age groups (12-14, 15-19, 20-24, 25-29, 30-34, and 35-39) are introduced as dummy variables, with the 25-29 age group taken as the reference category because it has the largest number of births and is therefore the most stable. The model is:

$$\text{Logit}\left[\text{F}\left(\text{i},\text{t}\right)\right]=\text{Constant}+\text{B}\times \text{Time}+\sum_{\text{i}}{\text{C}}_{\text{i}}\times {\text{X}}_{\text{i}}$$

where i is the age group and X_i _is the dummy variables associated with each age group, from 1 = 12-14, 2 = 15-19, etc. and 6 = 35-39, with the fourth group (ages 25-29) omitted as reference category.

This model allows one to compute age-specific fertility rates by period, to recalculate the cumulative fertility by age 40, and to estimate the trends. As in the first model, it provides a confidence interval for the slopes and allows simple testing for fertility stalls. This method requires only two basic hypotheses: homogeneity in risk of bearing a child over a short period of time and a constant age pattern of fertility over short periods of time, both of which appear realistic.

## Results

### Detailed example: Kenya, whole country

Both fitting methods were first applied to the case of Kenya, a country for which most authors agree that there was a fertility stall in the recent past. As expected, formal calculations demonstrate the fertility stall for the 1994 to 2002 period [see Table 1]. The demographic approach based on linear regression indicates no change of cumulative fertility by age 40 over the period 1994 to 2002, whereas fertility was declining fast before (from 7.42 to 4.66 from 1980 to 1994) and after (from 4.90 to 3.93 from 2002 to 2008). Both changes in slope, before and after 1994 to 2002, were highly significant (p < 10^-6 ^in both cases). Results based on the logistic regression (the statistical approach) confirm these findings with the same predicted values of cumulative fertility over the same periods, same trends, consistent slopes, and similar levels of significance.

**Table 1:** Comparison of two methods for fitting fertility trends and testing fertility stalls; Kenya, whole country

	Cumulative fertility TFR(40)		Time coefficient		Testing changing slopes		
		
Period	Begin	End	Slope	Std. error	T-test	p-value	Sample size
*Method 1: Linear regression*							*(Births)*
1980-1994	7.42	4.66	-0.19744	0.00769			47567
1994-2002	4.74	4.87	+0.01559	0.01446	13.010	< E-99	23575
2002-2008	4.90	3.93	-0.13854	0.02528	-5.293	1.2E-07	9900
*Method 2: Logistic regression*							*(Women)*
1980-1994	7.43	4.76	-0.04261	0.00131			17822
1994-2002	4.74	4.87	+0.00448	0.00280	15.215	< E-99	18970
2002-2008	4.92	3.92	-0.04001	0.00520	-7.526	5.3E-14	9713

Note: The TFR (cumulative fertility by age 50) can be derived from the TFR(40) by dividing by 0.90.

### Testing for fertility stalls in African countries

The same procedure was repeated for a number of situations where fertility stalls have been documented or suspected. Urban and rural areas were analyzed separately, because they were found to have divergent fertility trends [22]. Fertility stalls were found in the following: Ghana, urban areas in 1998-2008; Kenya, urban and rural areas in 1994-2002; Madagascar, urban areas in 1988-1994 and rural areas in 1988-1998; Nigeria, rural areas in 1988-1998; Rwanda, urban areas in 1989-1997 and rural areas in 1997-2008; Senegal, urban areas in 1995-2002; Tanzania, rural areas in 1996-2008; Zambia, rural areas in 1982-1997. In all cases, the fertility stall was well demonstrated by statistical testing, and the changes in slope from fertility decline to stall (and from stall to decline, when applicable) were highly significant in almost all cases but one, with p < 10^-3 ^[see Table 2]. The magnitude of the statistical evidence was comparable using either the linear regression or the logistic regression. The second approach provided a higher level of statistical significance in a majority of cases, although not in all cases.

**Table 2:** Comparison of two methods for testing fertility stalls in African countries

	Linear regression		Change	Logistic regression		Change
Period	Slope	Std. error	p-value	Slope	Std. error	p-value
*Ghana, urban*						
1975-1998	-0.10762	0.00608		-0.03269	0.00141	
1998-2008	+0.01426	0.01657	5.0E-12	+0.00344	0.00584	1.8E-09
*Kenya, urban*						
1963-1994	-0.12900	0.00684		-0.03333	0.00143	
1994-2002	+0.03509	0.02058	3.9E-14	+0.00821	0.00645	3.1E-10
2002-2009	-0.09650	0.03441	1.0E-03	-0.03026	0.01189	4.4E-03
*Kenya, rural*						
1980-1994	-0.19100	0.00866		-0.03968	0.00143	
1994-2002	+0.02426	0.01747	< E-99	+0.00627	0.00313	< E-99
2002-2009	-0.15017	0.03158	1.3E-06	-0.04220	0.00583	2.4E-13
*Madagascar, urban*						
1977-1988	-0.12079	0.03294		-0.03294	0.00683	
1988-1994	+0.04702	0.04199	1.7E-03	+0.01405	0.00910	3.6E-05
1994-2008	-0.09616	0.01129	9.9E-04	-0.03280	0.00308	1.1E-06
*Madagascar, rural*						
1977-1988	-0.07160	0.01914		-0.02444	0.00315	
1988-1998	-0.01631	0.01268	1.6E-02	-0.00076	0.00210	3.9E-10
1998-2008	-0.11494	0.01187	1.4E-08	-0.02714	0.00208	< E-99
*Nigeria, rural*						
1984-1988	-0.35712	0.04802		-0.07885	0.00790	
1988-1998	+0.04338	0.00700	2.2E-16	+0.00664	0.00136	< E-99
1998-2008	-0.13639	0.01458	< E-99	-0.03009	0.00236	< E-99
*Rwanda, urban*						
1982-1989	-0.27482	0.08527		-0.07242	0.01550	
1989-1997	+0.04975	0.03957	5.6E-04	+0.01618	0.00762	2.9E-07
1997-2008	-0.07676	0.02812	9.2E-03	-0.02420	0.00573	2.3E-05
*Rwanda, rural*						
1982-1997	-0.10730	0.00787		-0.02195	0.00129	
1997-2008	-0.01207	0.01344	9.7E-10	-0.00820	0.00236	3.2E-07
*Senegal, urban*						
1995-2002	-0.06199	0.01707		-0.01696	0.00416	
2002-2008	+0.03689	0.02256	4.7E-04	+0.00338	0.00570	3.9E-03
*Tanzania, rural*						
1977-1996	-0.04208	0.00635		-0.00775	0.00105	
1996-2008	-0.00127	0.01239	3.4E-03	-0.00153	0.00210	8.0E-03
*Zambia, rural*						
1968-1982	-0.05724	0.00845		-0.01047	0.00135	
1982-1997	+0.04599	0.02153	8.1E-06	+0.00763	0.00343	9.5E-07

### Checking for undocumented stalls

A number of fertility stalls were proposed by some authors but refuted by others [13]. These stalls were tested for countries as a whole, ignoring the urban/rural divide, as is usually done by other analysts. For our testing, the baseline date was chosen two years prior to the time at which the onset of the stall was proposed, since published estimates of TFR calculated over three years apply to the fertility level 1.5 years before the survey, on average. Among the seven countries investigated, none exhibited a significant stall during the proposed period when using the complete yearly data sets [See Table 3.] In Benin, Cameroon, and Mozambique, there were no significant changes in the slope of fertility declines since the previous surveys (2001 in Benin, 1998 in Cameroon, and 1997 in Mozambique), although the difference in slope was borderline when using the logistic regression in Mozambique. In Ethiopia and Uganda, the fertility decline accelerated during the second period (assumed to be a fertility stall), after 1999 in Ethiopia and after 1993 in Uganda. In Côte d'Ivoire and Zimbabwe, the speed of fertility decline was reduced significantly during the second period (1997-2005 and 1999-2006, respectively), but remained negative and far from a fertility stall defined by a slope equal to or above zero. In conclusion, none of these hypothesized stalls was found to be statistically significant, whether using a linear regression model or a logistic regression model.

**Table 3:** Testing for undocumented fertility stalls in African countries

	Linear regression		Change	Logistic regression		Change
Period	Slope	Std. error (slope)	p-value	Slope	Std. error (slope)	p-value
*Benin*						
1989-1999	-0.07096	0.01195		-0.01407	0.00141	
1999-2006	-0.03728	0.01739	0.110	-0.01131	0.00584	0.450
*Cameroon*						
1982-1996	-0.08245	0.01034		-0.02036	0.00176	
1996-2004	-0.06695	0.01929	0.708	-0.01846	0.00360	0.636
*Côte d'Ivoire*						
1985-1997	-0.19195	0.01293		-0.04394	0.00224	
1997-2005	-0.05228	0.02553	1.1E-06	-0.01767	0.00496	1.4E-06
*Ethiopia*						
1989-1999	-0.06771	0.00950		-0.01413	0.00157	
1999-2006	-0.12585	0.01898	6.2E-03	-0.03133	0.00339	4.1E-06
*Mozambique*						
1983-1995	-0.02685	0.01162		-0.00698	0.00202	
1995-2003	-0.04466	0.01629	0.373	-0.01388	0.00287	0.049
*Uganda*						
1974-1993	-0.03010	0.00751		-0.00699	0.00119	
1993-2006	-0.06326	0.01003	8.1E-03	-0.01484	0.00161	9.0E-05
*Zimbabwe*						
1989-1999	-0.14330	0.00751		-0.03537	0.00106	
1999-2006	-0.06095	0.01003	6.0E-07	-0.01914	0.00397	7.9E-05

## Discussion

In this exercise, we showed that two different methods led to basically the same conclusions with respect to fertility stalls and to equivalent slopes and confidence intervals. This is reassuring, since cumulative period fertility can be considered as a synthetic cohort or as the sum of age-specific fertility rates. This finding also indicates that basic assumptions underlying both methods are likely to be fulfilled, namely the equivalence of period and cohort and the constant age pattern of fertility during the process of fertility changes. What the preferable method of testing is can be discussed endlessly. The logistic regression method tends to provide lower p-values, which suggests that it is more sensitive. However, the linear regression method might be more specific and seems to provide a higher p-value when the case is borderline, therefore ignoring false positives (as seen for Mozambique over the period 1995 to 2003). Therefore, it may be more realistic. In any case, both methods led to the same conclusions in most instances.

Data quality is usually not an issue in DHS. Completed family size (or TFR) values tend to be very consistent for the same cohorts (or periods) in the same country, and comparisons with more precise data from demographic surveillance systems did not reveal errors that could not be explained by random fluctuations due to sample size and to cluster sampling [25]. Minor errors often found in DHS, such as the dating of birth around five years before the survey, are unlikely to affect levels or trends over longer periods of time; they will simply increase the variations in yearly estimates (i.e., data errors) and therefore reduce the power of statistical tests.

Other authors have used Poisson regression, or negative-binomial regression, to do similar testing of slopes. There is no obvious comparative advantage to using Poisson or negative-binomial regressions when calculating yearly fertility rates, since most women will have only one delivery at most over a 12-month period (the case would be different when using five-year periods or longer). Therefore, a simple outcome of 0/1 as used in the logistic regression method seems to be more appropriate. We did some basic comparisons of Poisson regression versus logistic regression and found similar results in the case of Kenya displayed in Table 1. Further testing could be done to investigate whether Poisson or negative-binomial regressions have any comparative advantage in this type of situation.

Other options are available for further testing fertility trends. For instance, one could use an age pattern of fertility in each situation in order to make the testing more precise. However, this is likely to be difficult. A Coale-Trussell function could be tried, but is likely to miss premarital fertility, which accounts for 20% to 40% of total fertility in some southern African countries [26]. Simpler functions such as polynomials could be tried, but probably with little advantage when compared with the straightforward empirical pattern presented by dummy variables associated with each age group.

Sophisticated statistical models have been developed in the past 20 years for testing changing slopes of a response variable in a variety of situations. Some of these models, such as "switching regression" or "change point regression" could also be tried to estimate fertility trends from DHS [27-29].

Using retrospective data leads necessarily to some minor mortality biases, compared with full-scale vital registration or prospective data. However, these biases are likely to be small, since mortality between age 12 and 40 is usually very low. In Africa, the high prevalence of HIV/AIDS and high mortality among young women could lead to larger biases. This would lead to overestimating fertility levels in recent years, since the fertility of HIV-infected women tends to be lower than that of others. In theory, this could produce some apparent fertility stalls in retrospective surveys, but in our studies we did not find any obvious correlation with HIV prevalence nor with HIV mortality. This point could be further investigated when more data become available.

Fertility stalls appear uncommon in African countries. Of the 31 countries investigated in our earlier studies, only eight exhibited some kind of fertility stall, of which five were restricted to either urban areas (Ghana and Senegal) or to rural areas (Nigeria, Tanzania, Zambia), while the other three cases affected both urban and rural areas (Kenya, Madagascar, and Rwanda). Most of these stalls were of short duration (< 10 years) or had been occurring for less than 10 years before the last survey. These stalls of short duration do not compare with formal stalls such as that of Argentina, which lasted for about 30 years (over an entire generation) at a much lower level of TFR (about three children per women). Fertility stalls in Africa appear so far to be minor accidents in the course of the fertility transition. However, if they last longer, they could have serious consequences for long-term demographic dynamics, especially when they occur at relatively high levels of fertility. Furthermore, African countries are still in the middle of the fertility transition, and anything could happen in the future. A recent study in the Pacific Islands showed that new forms of fertility stalls or of fertility reversals could happen as a result of deliberate reproductive strategies of couples. Because couples might have an economic advantage to produce children who will be sent later in migration and who could remit money to the family, they may choose to have more children [30].

Some of the fertility stalls proposed by other authors appeared undocumented in our analysis. This is due to the differences in case definition and in statistical testing. Using only two successive surveys with wide confidence intervals and point estimates based on a period of three years before a survey could be misleading when compared with a detailed analysis of fertility trends using all data available based on longer periods of 10 years or more. The case is even more delicate when comparisons are made on smaller sample sizes or when stalls are studied at the regional level or according to socioeconomic characteristics.

More research could be conducted on the rationale for these well documented stalls. In an earlier study, we showed that country situations were highly diverse, and one could use a variety of factors to explain them without any consistent pattern [23].

More research could also be conducted on the provision of family planning services, both in terms of quantity and in quality. Some authors have suggested that reduced financing for family planning services could explain the fertility stalls [31]. This could be further analyzed, case by case, while separating urban and rural areas whenever this is feasible.

## Competing interests

The author declares that they have no competing interests.
